# The potential roles of dental pulp stem cells in peripheral nerve regeneration

**DOI:** 10.3389/fneur.2022.1098857

**Published:** 2023-01-11

**Authors:** Jing Fu, Xigong Li, Feilu Jin, Yanzhao Dong, Haiying Zhou, Ahmad Alhaskawi, Zewei Wang, Jingtian Lai, Chengjun Yao, Sohaib Hasan Abdullah Ezzi, Vishnu Goutham Kota, Mohamed Hasan Abdulla Hasan Abdulla, Bin Chen, Hui Lu

**Affiliations:** ^1^Department of Stomatology, Affiliated Hangzhou Xixi Hospital, Zhejiang University School of Medicine, Hangzhou, China; ^2^Department of Orthopedics, The First Affiliated Hospital, College of Medicine, Zhejiang University, Hangzhou, Zhejiang, China; ^3^Oral and Maxillofacial Surgery Department, The Second Affiliated Hospital of Zhejiang University, Hangzhou, Zhejiang, China; ^4^Zhejiang University School of Medicine, Hangzhou, Zhejiang, China; ^5^Department of Orthopedics, Third Xiangya Hospital, Central South University, Changsha, Hunan, China; ^6^Alibaba-Zhejiang University Joint Research Center of Future Digital Healthcare, Zhejiang University, Hangzhou, Zhejiang, China

**Keywords:** dental pulp stem cells, neuron, Schwann cells, peripheral nerve diseases, neurotrophic factors

## Abstract

Peripheral nerve diseases are significantly correlated with severe fractures or trauma and surgeries, leading to poor life quality and impairment of physical and mental health. Human dental pulp stem cells (DPSCs) are neural crest stem cells with a strong multi-directional differentiation potential and proliferation capacity that provide a novel cell source for nerve regeneration. DPSCs are easily extracted from dental pulp tissue of human permanent or deciduous teeth. DPSCs can express neurotrophic and immunomodulatory factors and, subsequently, induce blood vessel formation and nerve regeneration. Therefore, DPSCs yield valuable therapeutic potential in the management of peripheral neuropathies. With the purpose of summarizing the advances in DPSCs and their potential applications in peripheral neuropathies, this article reviews the biological characteristics of DPSCs in association with the mechanisms of peripheral nerve regeneration.

## 1. Introduction

Peripheral neuron degeneration, inflammation, and necroptosis caused by trauma, diabetes, and neurodegenerative disorders may cause motor-sensory dysfunctions (1–3). Therefore, the current therapeutic regimen mainly focuses on neuron regeneration and function restoration in post-traumatic events.

Conventional therapies have limited efficacy in restoring nerve function since the regeneration of neurons, and glial cells require sufficient neuronal precursor cells, which are absent or lacking in the mature nervous system (4). Stem cell-based therapies bring new insight into the biotherapy of peripheral neuropathies, providing adequate cell sources capable of self-renewal and multi-directional differentiation (4–8). However, challenges remain in the mass production of autografts or autologous cells for sufficient nerve regeneration (5–8).

Derived from the neural crest, DPSCs yield great potential in differentiation into neurons, expression of various neurotrophic factors for axonal regeneration, and functions of immunomodulation, indicating that DPSCs are an ideal cell source for peripheral nerve regeneration (5–7). In this review, we will summarize the biological characteristics of DPSCs and their respective application in animal models of peripheral neuropathies, with a focus on their regenerative mechanisms for future application.

### 1.1. Biological characteristics of DPSCs

Dental pulp, identified as a typical soft tissue, is rich in blood vessels, nerves, and mesenchymal tissue. Dental pulp has a central role in the development of primary and secondary teeth and further maintenance throughout life (7, 9, 10). Gronthos et al. (11) first described that DPSCs were initially discovered from the third molar dental pulp, which is later found in other dental pulps including deciduous teeth, permanent teeth, and supernumerary teeth. DPSCs display fibroblast-like morphology with higher proliferation capacity but lack specific surface biomarkers (Table 1) (12–20). While highly express MSC-like phenotypic biomarkers including CD29, CD90, and CD73 (12, 13), DPSCs are also found to express stemness-related markers such as Oct-4, Nanog, and Sox-2 (14, 15), and cytoskeleton-related markers such as Nestin and Vimentin (15). Moreover, several studies have demonstrated the expression of cranial neural crest cell-related neural markers by DPSCs, including glial fibrillary acidic protein (GFAP), β-III tubulin, and microtubule-associated protein-2 (MAP-2) (15, 16, 21). Recently, several special markers are proposed to distinguish DPSCs from gingiva-derived mesenchymal stem cells (GMSCs) including Calreticulin, Annexin A5, and Rho GDP dissociation inhibitor alpha (17). Furthermore, a recent study by Lei et al. (18) demonstrated that the CD271 is the most effective stem cell surface marker for dental mesenchymal stem cells (DMSCs), which display high odontogenic potential.

**Table 1:** The features of DPSCs.

**Type**	**Surface markers expression**
MSC-like phenotypic markers	CD27, CD29, CD44, CD73, CD90, CD105, CD146, CD166, CD271, and STRO-1
Stemness-related markers	Oct-4, Nanog, and Sox-2
Cytoskeleton-related markers	Nestin and Vimentin
Neural crest cell-related markers	GFAP, β-III tubulin, and MAP-2
DPSC Specific markers	Calreticulin and CD271

## 2. The potential mechanisms of DPSCs in neural regeneration

**Figure 1:**
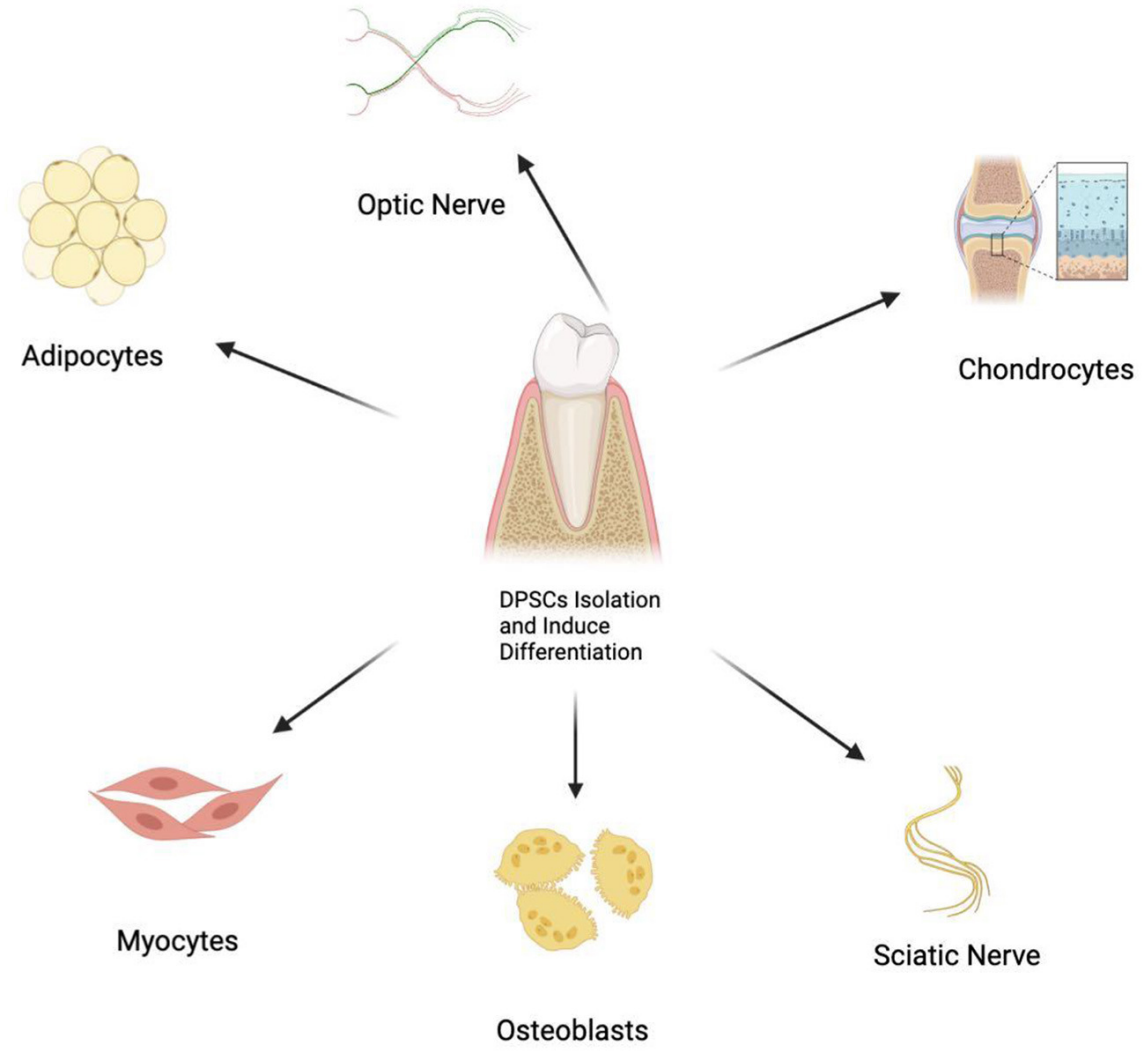
Multiple differentiation potential of DPSCs into various cell types.

**Figure 2:**
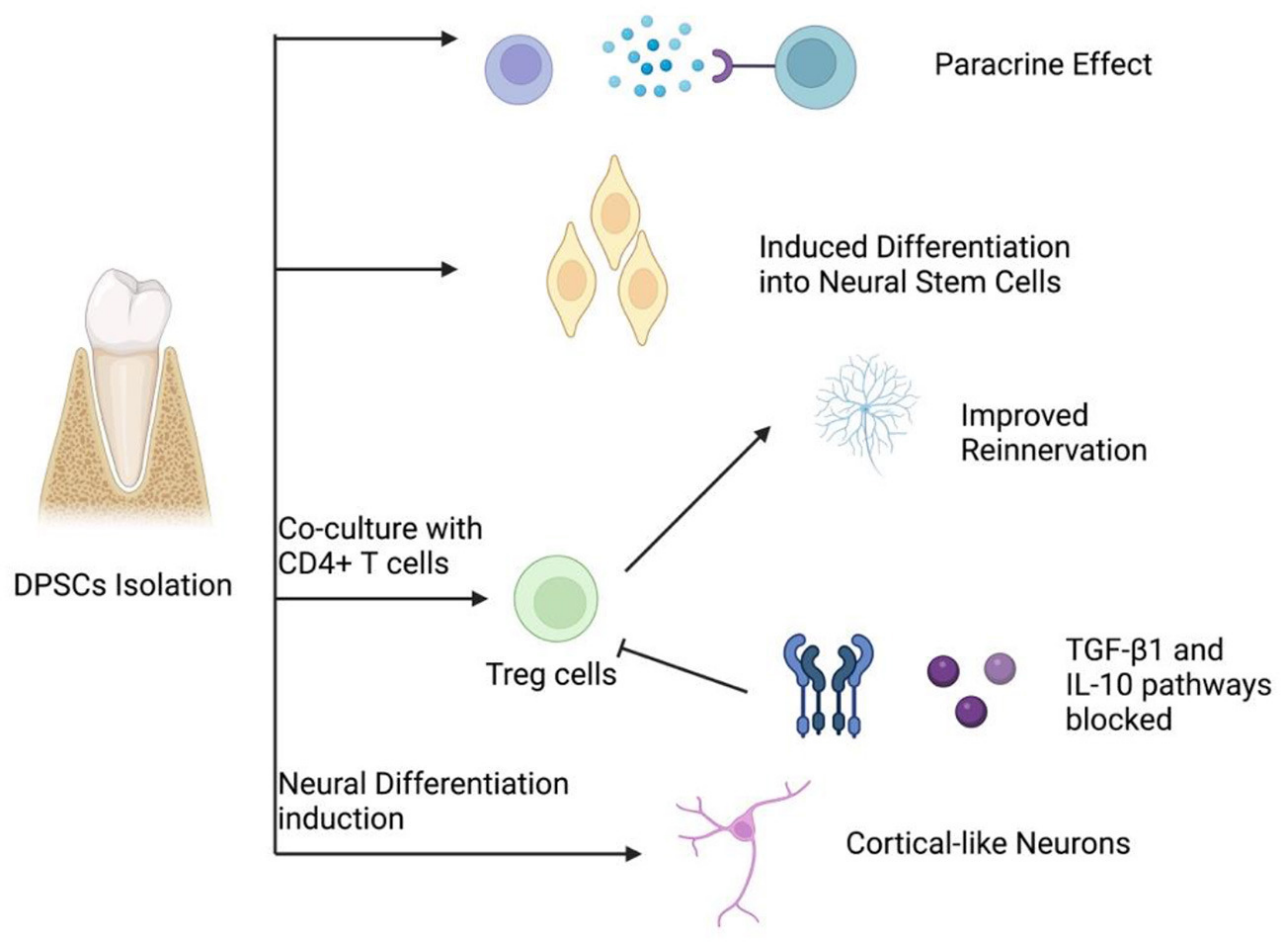
Four main mechanisms of peripheral nerve regeneration by DPSCs.

DPSCs have shown the potential of multiple differentiations, with promising therapeutic value as bioengineered autografts for different tissue repair (Figure 1). Therefore, DPSCs can be applied in the biotherapy for a variety of peripheral neuropathies (5–7, 19, 20, 22–26). Studies show that DPSCs could induce restoration in peripheral nerves *via* three mechanisms, which include neuronal differentiation, paracrine and immunomodulatory effects (Figure 2) (5–7, 19, 20, 22–26).

### 2.1. Neuronal differentiation

DPSCs can directly differentiate into neuron-like cells and express early neural markers (such as Nestin) (15). These neuron-like cells could migrate to the lesion sites and subsequently participate in the nerve regeneration process (5–7). Some studies have also shown that during migration, the transplanted DPSCs can recruit endogenous neural stem cells for tissue reconstruction (20). In addition, Kiraly et al. (15) revealed that the induction and differentiation of DPSCs are promoted by activating the intracellular cyclic adenosine phosphate signaling pathway. Chen et al. (27) proposed that the elevated intracellular cyclic adenosine phosphate can activate protein kinase A (PKA), which then up-regulate regeneration-related genes, such as arginase I, and promotes peptide synthesis. On the other hand, PKA also inhibits the Rho protein activation induced by myelin and induce nerve regeneration. Heng et al. (28) suggest that EphrinB2 signaling can modulate the neural differentiation of DPSCs, while EphB4 -inhibition in DPSCs could significantly up-regulate expression of the neural markers microtubule-associated protein 2, Musashi1, NGN2, and neuron-specific enolase. In 2018 Urraca et al. (29) described DPSC-derived neurons expressing GABAA and MAP2 genes, which is previously absent in undifferentiated DPSC and may provide future neurogenetic research with a useful tool.

### 2.2. Paracrine effects

Several studies have indicated that DPSCs could participate in the process of nerve repair in a paracrine manner (10, 20, 30). DPSCs can express brain-derived neurotrophic factor (BDNF), glia cell-line derived neurotrophic factor (GDNF), NGF, and neurotrophin-3 (NT3) at a substantial level, and exert neuroprotective functions in the process of peripheral nerve regeneration (10). Some researchers propose that the effects of these neurotrophic factors on neural cells are achieved *via* the intervention of PI3K/AKT signaling pathway. Even in the presence of neurotrophic factors such as NGF and GDNF, inhibition of PI3K or AKT activation can result in neuron apoptosis or necroptosis (31). The Erk signaling pathway plays a essential role in axonal growth stimulation by modulating neurotrophic factors (32). Additionally, the neurotrophin expression in DPSCs isenhanced in certain neural inductive conditions (33). The conditioned medium of DPSCs has been shown to increase Schwann cell proliferation rate while inducing neurite growth *in vitro* (31, 32). Previous studies revealed that the DPSC-derived secretome or DPSC conditional medium, which includes soluble factors and extracellular vesicles, proved to be therapeutically relevant in the management of neurodegenerative disorders and nerve injuries *via* the regulation of several processes, including neuroprotection, anti-inflammation, anti-apoptosis, and angiogenesis (34).

### 2.3. Immunomodulatory properties

The immunomodulatory properties of DPSCs may also exert crucial functions during the neural repair mechanism (35–38). Several studies have shown not only CD4+T cells co-cultured with DPSCs can highly express regulatory T cells (Treg), but DPSCs implanted *in vivo* could reverse the decrease in Treg expression induced by transforming growth factor β1 (TGFβ1) and interleukin-10 signaling pathways inhibition, suggesting that DPSCs could interfere with immunoregulation during nerve regeneration (35, 36). On the other hand, it has been discovered that DPSCs may inhibit TNF-α, and thus up-regulate anti-inflammation cytokines and promote nerve regeneration (37). furthermore, DPSCs can induce inhibition in cytotoxic T cell proliferation and activation *via* CD73, a central enzyme in the crosstalk of immunosuppressive adenosine and extracellular pro-inflammatory ATP, which is highly expressed in DPSCs (38).

## 3. The treatment of transplanted DPSCs in peripheral nerve disease

### 3.1. Peripheral nerve injuries

**Figure 3:**
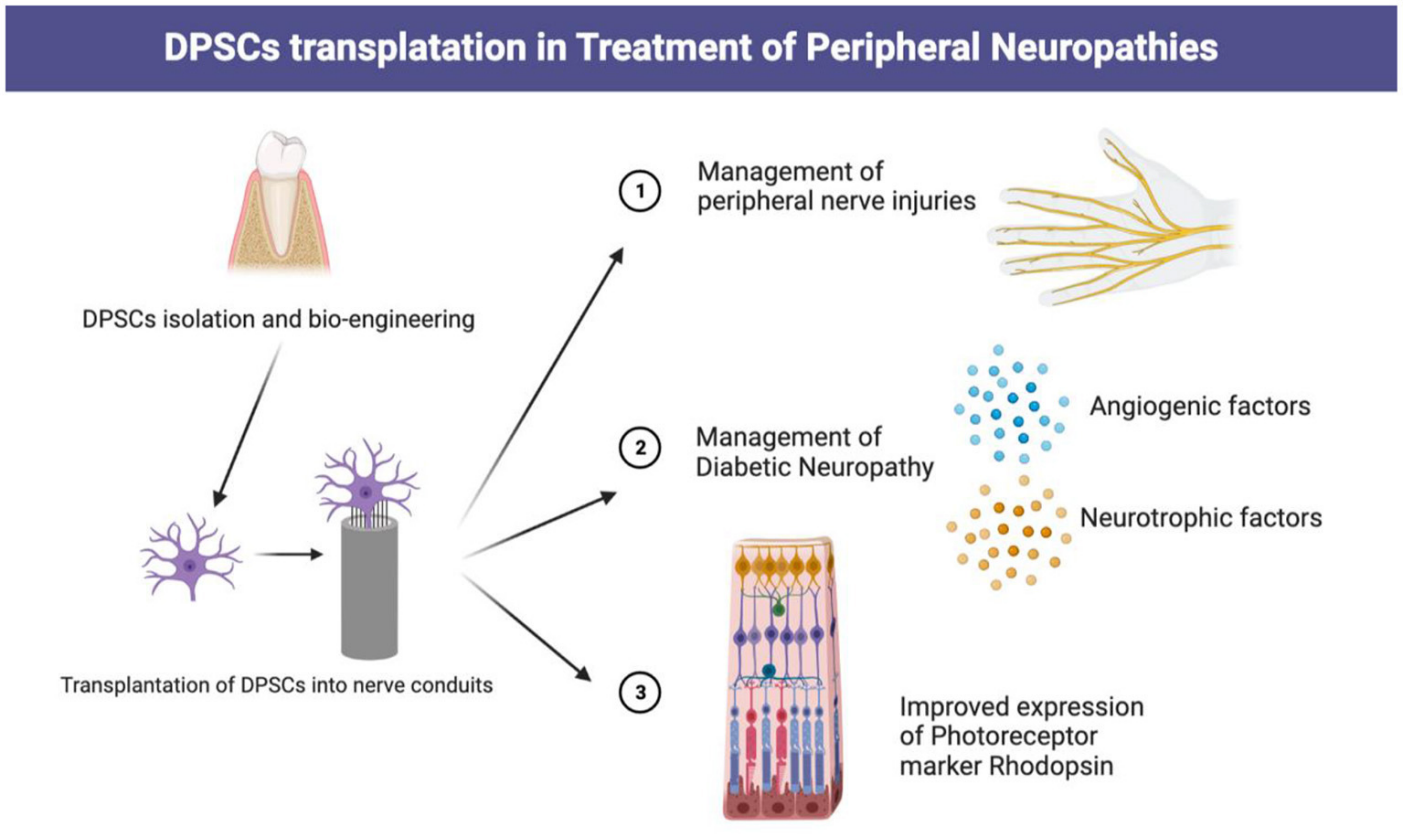
Transplantation of DPSCs in management of various peripheral neuropathies.

Peripheral nerve injuries lead to notable functional impairments and decreased quality of life (39). Despite advances in microscopic techniques for neurosurgery, clinicians always desire to improve postoperative nerve regeneration and rehabilitation for better functional restoration *via* various methods including biotherapy (40, 41). Currently, the transplanted DPSCs alone or in combination with several novel nerve conduits are promising therapy for patients suffering from peripheral nerve injury (42, 43). Takaoka et al. (44) transplanted DPSCs into a rat model with a 10-mm sciatic nerve defect and found improvement in axon growth, remyelination, electrophysiological activities, and alleviated muscle atrophy at 12 weeks post-transplantation. DPSC-embedded polymeric biomaterial based on ethyl acrylate and hydroxy ethyl acrylate copolymer shows sufficient bioactivity to promote regeneration of the injured sciatic nerve (45). A similar study has shown that preloading collagen conduits with Schwann cell-like cells (SCLCs) induced from DPSCs could enhance sciatic nerve repair (46). In addition, collagen scaffolds preloaded with DPSCs post-differentiation could exhibit certain traits of SCLC that promote the outgrowth of axons and myelination in 2-dimensional or 3-dimensional culture conditions (47). These results demonstrate that DPSCs are excellent stem cell sources for peripheral nerve regeneration (Figure 3).

### 3.2. Diabetic neuropathy

Diabetic neuropathy, the most common complication of type 1 and type 2 Diabetes Mellitus (DM), and has become a substantial health concern worldwide, especially for the elderly. It has been estimated that over 50% of long-term DM patients will eventually develop neuropathy, which could lead to diabetic foot ulcers associated with serious disabilities (48). The COVID milieu since 2019 has driven healthcare professionals to emphasize the matter of management and nursing of patients suffering from diabetic neuropathy since DM patients are prone to have compromised immunity and disturbed microenvironment (49). Typically, patients suffering from diabetic neuropathy show decreased peripheral nerve vascularity and a deficiency of angiogenic and neurotrophic factors, which may account for the pathogenesis of neuropathies (50, 51). Recently, the therapeutic effects of DPSCs in diabetic neuropathy have become recognized by many researchers, which raised controversies regarding the optimal application method of DPSCs. Makino et al. suggested that DPSCs transplantation can significantly improve the blood flow, nerve conduction velocity, capillary density, and intra-epidermal nerve fiber density of the damaged nerves while up-regulating the expression levels of angiogenic and neurotrophic factor genes (52). Another study proved that transplanted-DPSCs can significantly reduce the number of macrophages in the diabetic peripheral nerve microenvironment and specifically inhibit M1 macrophage expression while up-regulating M2 macrophage expression, eventually decreasing the M1/M2 macrophage ratio (19). Apart from regulating macrophage expression, DPSCs could also exert anti-inflammatory effects *via* inhibiting tumor necrosis factor α (TNFα) and interleukin-6 (IL-6) expression while up-regulating TGF-β expression. These findings provide some perspectives on possible future applications of DPSCs in diabetic neuropathy management.

### 3.3. Retina injury

Neurons in the retina and optic nerve share a mutual origin from the embryonic diencephalon. After neuroepithelium formation of the retina, neurons lose the ability to divide, rendering neuronal renewal in the retina impossible (53). Therefore, blindness caused by retinal injuries remains a major cause of disability worldwide. Retinal ganglion cells express a large number of neurotrophic factor receptors, which may enhance retinal ganglion cell survival and axonal regeneration (54). Mead et al. found that DPSCs secreted a large number of neurofibrillary tangles (NTF), which enhanced neural βIII-tubulin+ retinal cell proliferation and lengthened the neuritis (55). In addition, transplantation of DPSCs into the vitreous humor of mice after optic nerve injury promoted Brn-3a+ retinal ganglion cell survival and axonal regeneration (55). It has been reported that 44% of DPSCs expressed a photoreceptor marker rhodopsin in a conditioned medium from the damaged retina (56). This promising novel mechanism should be further explored for clinical applications.

## 4. Conclusions

### 4.1. Current research and challenges

Dental pulp tissue yields great reproductive ability and is rich in varying categories of stem cells with unique differentiation potentials. It has been concluded that DPSCs may be isolated from both postnatal teeth and extremely rare natal teeth (11). Immortalized DPSCs are also an excellent source of pluripotent stem cells with high molecular, morphological and genetic resemblance with non-immortalized DPSCs, which introduces the possibility of building a reservoir with immortalized DPSCs from patients suffering from a wide spectrum of neurogenetic disorders (57). On the other hand, Wilson et al. (58) have assessed the tumorigenic potential of immortalized DPSC *in vitro* and in mice and observed no tumor formation, indicating the probable safety of immortalized DPSC in future clinical applications.

As presented in Table 2, current research on the application of DPSC in the management of central and peripheral neuropathies is majorly concentrated on differentiation induced *in vitro* and implantation either *via* nerve conduit scaffold or direct injections. However, for such a therapeutic regimen to be carried out clinically, further clinical and lab research are required to achieve large-scale DPSC manufacture, storage, and transportation with minimum possibility of contamination. Concerns have been raised about the compromised quality of DPSC culture possibly in association with poor oral hygiene and long-distance transportation (57).

**Table 2:** Several current studies involving the application of DPSCs in nerve regeneration.

**No**.	**Method/treatment of DPSCs**	**Study type (*in vitro* versus *in vivo*)**	**Biomarkers expressed by DPSCs**	**Outcome**	**Reference**
1	Nerve conduit preloaded with DPSCs	*In vivo* rat sciatic nerve	GFAP and β-tubulin III	Promote functional repair of peripheral nerve injuries	(59)
2.	Hypoxia-treated DPSCs transplantation into damaged rat spinal cord	*In vivo* rat spinal cord	CD13+, N-cadherin and bFGF	Increase vascularization and oxygenation of the injured spinal cord	(60)
3.	5-Aza pre-condition to induce myogenic commitment; injection into rat urethral sphincter post pudendal nerve transection	*In vitro* and *in vivo* rat urethral sphincter	HLA-ABC	*In vitro*: DPSCs committed toward myogenic lineage; *In vivo*: promoted vascularization, recovered sphincter thickness and detected within the nerve	(61)
4.	DPSC auto-transplantation in unilateral hindlimb of diabetic rats	*In vivo* in diabetic rat sciatic nerve	CD29, CD34, CD49d, CD45 and CD90	Improved blood flow, nerve conduction velocity, capillary number, and intra-dermal nerve fiber density	(62)
5.	Exposure of DPSCs to midbrain cues	*In vitro*	CD73, CD90, CD105, CD34, and HLADR	Midbrain cues could dictate DPSCs to dopaminergic cell-type	(63)
6.	DPSCs pre-labeled with PKH 26 is cultured and injected intravenously in PD rat model	*In vivo* in PD rat model	CD34, CD45, CD73, CD90, CD166, and HLA-DR	Amelioration of degenerated neurons, and enhancement to impaired behavioral performances	(64)

GFAP, glial fibrillary acidic protein; bFGF, basic fibroblast growth factors; HLA-ABC, Human Leukocyte Antigen–A, B, and C isotype; HLA-DR, Human Leukocyte Antigen –DR isotype; PD, Parkinson's disease.

### 4.2. Future prospects

Stem cell-based therapies shed light on the biotherapies of peripheral nerve disease. DPSCs may enhance peripheral nerve regeneration *via* the induction of neuronal differentiation and the up-regulation of various neurotrophic factors. DPSCs in combination with biomaterials could be the prospect of neural tissue repair. Furthermore, DPSCs have a wide range of application prospects in peripheral nerve diseases, such as peripheral nerve injury, diabetic neuropathy, and retina injury. While DPSCs transplantation shows promising therapeutic potential in the management of peripheral nerve diseases, further research is required to establish a therapeutic approach and a regimen of dosage, efficacy, and safety. In conclusion, DPSCs yield great potential in peripheral neural tissue regeneration and repair, yet various issues remain to be solved through further assessment and experimentation.

## Author contributions

HL and JF designed the study. FJ, XL, and YD performed data collection. HZ, AA, ZW, and JL analyzed the results. CY, SE, VK, BC, and MH drafted the manuscript. All authors have read and approved the final manuscript.
